# Regional Assessments Under the Canadian *Impact Assessment Act:* Objectives, Outcomes and Lessons So Far

**DOI:** 10.1007/s00267-025-02176-4

**Published:** 2025-05-14

**Authors:** Steve Bonnell

**Affiliations:** Impact Assessment Agency of Canada, and Department of Geography, Memorial University of Newfoundland, St. John’s, NL Canada

**Keywords:** Regional assessment, Canada, Objectives, Outcomes

## Abstract

The planning and conduct of regional assessments (RAs) under the Canadian *Impact Assessment Act (*IAA) has reflected various objectives and planned outcomes. To date, this has included a key focus on improving the effectiveness and efficiency of subsequent project assessments through RA-provided information, analysis and mitigation, although the manner and degree to which these outputs will transfer to and affect the scope of later assessments has yet to be confirmed. Some RAs have also been designed to provide larger effects management and planning outputs, including identifying and recommending broader initiatives for addressing effects and maximizing benefits from future development. RA’s potential role in influencing the nature, intensity and distribution of future activities has also been recognized, although this can be challenging where there is no regional planning mechanism for RA to engage with, and especially, given Canadian jurisdictional realities. RAs under the IAA are most likely to be successful in that regard where they are designed and conducted in cooperation with other jurisdictions, and especially, have a direct link to existing and applicable planning processes. Experience also suggests that even where this is the case, there may be challenges if neither process establishes an overall vision for future development, or where there is a lack of specificity in RA outputs or how they are planned to be used in decision-making.

## Introduction

Impact assessment (IA) is an important and widely used process for identifying the potential effects of proposed development activities, and for considering and addressing these in decision-making^1^. IA has traditionally and primarily been applied to individual development proposals (Morgan 2012), and despite its at times demonstrated or perceived utility in improving project designs and decisions (Cashmore et al. 2004; Gronow 2022), the limitations of its inherently reactive nature and focus on project-specific effects and decisions have also been recognized (Dalal**-**Clayton and Sadler 1999; Stinchcombe and Gibson 2001; Tetlow and Hanusch 2012). This has prompted growing interest in broader and more proactive applications of IA principles and approaches, including the upstreaming (Jay 2010) and upscaling (Noble and Hanna 2015) of IA use through its application to earlier stages of planning and across sectors and regions. Globally, this is reflected in the establishment of strategic environmental assessment (SEA) systems in many jurisdictions (Tetlow and Hanusch 2012; Nwanekezie et al. 2022) and their application to a variety of planning processes and associated policies, plans, programs (PPPs) and other strategic initiatives. Other “beyond-project” IA approaches have seen comparatively less advancement of theory or evidence of practice, including regional assessments (RAs) of past, present and potential development activities within a defined geographic area (Arnold et al. 2022a).

In Canada, well-established IA processes and decades of experience at the project level have likewise created interest in the adoption and use of higher-order IA approaches. Notwithstanding examples of strategic, regional and other such assessments undertaken in various jurisdictions throughout the country (Noble et al. 2019; Blakley et al. 2020; Noble 2021), their completion and use under federal IA legislation is a somewhat more recent phenomenon. The Canadian *Impact Assessment Act* (IAA), which came into force in 2019, includes provisions for the planning and conduct of both RAs and strategic assessments (SAs^2^) and requirements for their consideration in subsequent project IAs. Although this legislation has been in place for only just over five years, it is notable that most of the discussion and almost all practice under the IAA thus far has been in relation to RA rather than SA.

The RA provisions of the IAA are quite general and largely procedural in nature, providing considerable flexibility in the planning and conduct of individual assessments. This is often seen as a key strength (Doelle and Critchley 2015) as it allows each RA to be designed fit-for-purpose and thus to address the specific issues, requirements and circumstances that characterize it. At the same time, this flexibility along with widely varying perspectives amongst RA participants around what they should be and do, how they should be conducted, and what they should assess and ultimately deliver, has seen a range of objectives and outcomes reflected in RA discussions and practice thus far (Bonnell 2024). There have been calls for continued flexibility in RA design to address contextual requirements, as well as for adherence to fundamental principles and best practice (Gibson et al. 2020).

A review of RA experience under the IAA over these initial five years is therefore in order, to gain insights and derive lessons that may help inform future RA planning and conduct in the Canadian federal context, as well as make associated contributions to the advancement of RA theory and practice in general. This paper provides such a review, and addresses the following research questions:

1. What have been the primary objectives and intended outcomes^3^ of the various RAs that have been requested, completed or are currently underway under the IAA?
2. How do these relate to the potential RA objectives and outcomes being referenced in the theoretical literature?
3. Are there any key determinants of these aspects of RA design and conduct under the IAA, including any identifiable enablers or challenges to establishing and achieving certain RA objectives or outcomes in practice?

In doing so, it explores opportunities for future advancement and improvement in RA practice under the IAA, in order to help ensure that such assessments live up to their full potential while also reflecting goals and expectations that are grounded in the requirements and realities of Canadian federal IA legislation, processes and jurisdiction.

## Theoretical Context: RA Definition, Rationale, Objectives and Outcomes

The following sections provide a brief overview of the RA literature, including current theoretical perspectives around RA’s definition, rationale, objectives and potential outcomes, as background and context for the subsequent evaluation of RA practice under the IAA.

An early RA definition was provided by Goodland and Tillman (1995: 13, cited in Annandale et al. 2001), who framed it rather simply as a “process of determining the regional environmental implications of multi-sector developments within a defined geographic area over a certain time period”. The array of possible RA goals and approaches that have been put forward by IA theorists, practitioners and participants in the years following, however, has made a single definition that captures all of these dimensions somewhat elusive (Arnold et al. 2022a). What is clear is that RA and other higher-order IA approaches were originally conceived as a means of addressing the long-recognized limitations of project-specific assessments (Gunn and Noble 2011; Noble et al. 2017; Arnold et al. 2022a) – including their inability to effectively address larger issues that require early and regional-scale analysis, as well as approaches to their management that go beyond individual project designs, mitigation and decisions (Fidler and Noble 2013). This includes cumulative effects resulting from past, present and foreseeable projects and activities (Duinker et al. 2013; Sinclair et al. 2017; Blakley and Noble 2021), the analysis and management of which are typically beyond the ability and/or responsibility of individual project proponents, assessments and decisions.

It has been noted that RAs may be useful when a relatively pristine area is being proposed for development (Annandale et al. 2001; Fidler and Noble 2013), or where there are concerns about past effects and a desire to avoid or reduce further contributions to these or even to remedy past damage (Montour et al. 2022). RA need can be triggered by a single large development proposal in an area (Annandale et al. 2001), including those that may induce future activities and effects (Bonnell et al. 2002; Olagunju and Gunn 2015; Johnson 2016; Johnson et al. 2020), or when multiple developments are planned or anticipated. Several RA types and focus areas have also been identified, and they may consider activities in a single sector (World Bank 1996; Davey et al. 2002; Baker and Kirstein 2012) or developments of multiple types and scales (Rees 1999; Harriman and Noble 2008; Rathi 2021). RAs can focus on a single, important environmental component or issue (Lloyd Jones et al. 2015; Blakley et al. 2020), or they may be multi-dimensional and integrative by considering a variety of environmental, social, economic, cultural and health effects (Doelle and Critchley 2015; Halseth et al. 2016; Atlin and Gibson 2017; Buse et al. 2020; Arnold et al. 2022b).

Various potential objectives for, and intended outcomes of, RAs have also been suggested in the literature and reflected in practice to date (Arnold et al. 2022a). These range from helping inform, and improve the effectiveness and efficiency of, subsequent project IAs (World Bank 1996; Rees 1999; CCME Canadian Council of Ministers of the Environment (2009); Gunn and Noble 2009a, b; Franks et al. 2010; Lloyd Jones et al. 2015; Noble 2017) to more planning-oriented outputs. The latter can include identifying alternative development scenarios and evaluating these against regional capacities, goals and values (Harriman and Noble 2008; Gunn and Noble 2009a, b; Atlin and Gibson 2017) to help influence the nature and pace of future development (Noble 2008; Gunn and Noble 2009a). These have been envisioned along an “assessment/management/planning continuum” (Bonnell 2024) (Table 1), which suggests that these can range from outputs that focus solely on informing future project IAs, to assessing and evaluating effects on a regional scale, to engaging with larger regional effects management approaches and planning initiatives, along with some “straddling” outputs that extend across several of these dimensions. Each of these is described briefly in the sections that follow.

**Table 1:** An assessment/management/planning continuum of potential RA objectives and outcomes

### Informing and Improving Project IAs

#### Environmental Information

RAs can be a means of identifying, compiling and presenting existing and available information from a variety of sources. This can be used to provide an understanding and description of the existing environment of a region (Davey et al. 2002; Horvath and Barnes 2004; Johnson et al. 2011), including past, current and foreseeable development activities that have or may influence these conditions. It can also help identify important information or knowledge gaps, and possibly address these through primary data collection or recommendations for future studies (World Bank 1996). A key objective is often to make such information available for use in future IAs, thereby improving their overall effectiveness and efficiency. It has been suggested, however, that a compilation of environmental information does not in itself constitute an RA, which must involve some form of analysis of past or potential developments and effects (Arnold et al. 2022a).

#### Potential Effects and Mitigation

RA analysis and engagement can also help identify key environmental issues and concerns related to past, proposed or potential development activities in a region. This can again inform subsequent project assessments (Horvath and Barnes 2004, Gunn and Noble 2015), including helping to better scope these IAs to focus on issues that require detailed consideration at the individual project level. RAs can also identify and recommend mitigation and follow-up requirements for future projects (Horvath and Barnes 2004, Braun 2008; Thorne et al. 2009; Gunn and Noble 2015). This can include assembling, evaluating and potentially codifying standard measures and requirements, as well as new or refined measures to address key gaps or for emerging industries, again with implications for the effectiveness and efficiency of later IAs.

### Regional Effects Analysis, Management and Planning

Despite continued interest in RAs as a means of informing subsequent project IAs, it has also been suggested that they should not just be about scaling-up traditional IA principles and approaches to the regional level (Gunn and Noble 2009b, 2015; Arnold et al. 2022a), nor simply integrating RA outputs into project IA practice. There is clear overlap here, however, as regional information, context and analysis provided by an RA can be used to inform both project IAs (as discussed above) and regional planning activities (Table 1). Also, resolving complex regional issues through RA can itself have implications for downstream IA efficiency by helping remove them from project-level reviews (Gibson et al. 2010).

#### Regional Effects Analysis and Context

RA can provide a mechanism to proactively, comprehensively and simultaneously consider all relevant activities and influences, over appropriate spatial and temporal scales (Noble et al. 2017; Sinclair et al. 2017; Blakely and Russell 2022), including how multiple developments interact with each other and the environment (Doelle and Sander 2020). In doing so, RA information and analysis can provide “regional context” for future projects and assessments (Noble 2005; Franks et al. 2010; Fidler and No**b**le 2012; Gunn and Noble 2015; Arnold et al. 2022a), or as input to regional planning initiatives. This can include identifying important or sensitive environmental features (Lloyd Jones et al. 2015), such as locations that are particularly vulnerable to further disturbance due to accumulations of past activities and effects. This can be useful in future IAs by helping evaluate future project proposals or development scenarios in context (Horvath and Barnes 2004), including their relationship and potential contributions to regional cumulative effects.

#### Going Beyond Project-Level Mitigation

RA can provide a forum to identify solutions to important, regional-scale issues (Davey et al. 2002; Gunn and Noble 2009b; Johnson et al. 2011) that go beyond project-level mitigation (Arnold et al. 2022a). This can include programs and initiatives by governments or other parties to address the effects of past or anticipated development activities, such as new regulations and increased enforcement to help manage induced actions and effects following new road access to previously remote areas (Bonnell et al. 2002). It can also involve identifying and recommending required improvements in the availability or capacity of public services and infrastructure to address anticipated population growth from upcoming developments, measures to create or maximize social and economic benefits, or others that enhance institutional capacity or foster innovation and collaboration (Blakely et al. 2020). As any such initiatives would be beyond the ability and responsibility of individual projects and proponents, an RA can provide a mechanism for early identification of issues and requirements, and for making associated recommendations to the appropriate responsible authorities.

#### Influencing Regional Planning and Decision-making

Arguably the most proactive, comprehensive and thus effective approach to addressing regional environmental issues is by influencing the overall nature, intensity and distribution of development activity in the area in question. RA can, for example, help create overall guidance or consent rules for future projects (Therivel and Ross 2007; Baker and Kirstein 2012), such as requirements to avoid environmentally sensitive areas or to implement additional mitigation in certain situations (Gunn and Noble 2009b). Deeper engagement with regional planning has also been envisioned, where RA can identify and evaluate alternative development scenarios based on regional capacities and thresholds (Davey et al. 2002; Horvath and Barnes 2004; Gunn and Noble 2009a, 2015; Johnson et al. 2011; Olagunju and Blakley 2017; Blakley et al. 2020; Johnson and Ray 2021; Pope and Young 2024) or socially derived and context-specific targets, goals and values (Harriman and Noble 2008; Gunn and Noble 2009a, 2009, b, 2015; Franks et al. 2010; Arnold et al. 2022a), with an emphasis on achieving sustainability (Braun 2008; Gunn and Noble 2015; Atlin and Gibson 2017).

While a regional planning emphasis is not necessarily required (Arnold et al. 2022a) or yet common (Blakely et al. 2020) in RA practice, and there is continued discourse around the degree to which RA should itself emulate planning or retain its separate “assessment” function (Gunn and Noble 2011; Gibson et al. 2020), an important focus of the current RA literature is on its potential to in some manner influence the nature and pace of development (Gunn and Noble 2009a).

## RA in Canada

Despite being at a relatively early stage of practice at the federal level in Canada, RAs or RA-like initiatives have been undertaken in parts of the country for decades. Blakely et al. 2020 identified and reviewed 42 Canadian RAs completed between 2000 and 2020, finding that many have been conducted voluntarily, primarily by governments but with examples of Indigenous-led assessments and others involving multiple parties. There has thus far been a concentration of RA practice in western and northern Canada, with examples of both single- and multi-sectoral RAs and considerable variability in RA rationales, goals, outputs and approaches. The majority of the cases reviewed (88 percent) included a significant focus on cumulative effects, and were considered “strategic” (67 percent) in that one of their chief aims was to influence the nature of future development in the area (Blakely et al. 2020). That research did not, however, summarize or evaluate the specific objectives of the RA case studies reviewed, and as only one RA had been completed under federal IA legislation at that time, there was limited analysis of RA experience under the IAA.

### RA Under Canadian Federal IA Legislation

Although federal IA in Canada has primarily been a project-by-project endeavor, regional-scale initiatives have been at least referenced in all associated legislation to date. The first such statute, the *Canadian Environmental Assessment Act* (1992), while not specifically contemplating RA conduct under that legislation, allowed that:

16.2 The results of a study of the environmental effects of possible future projects in a region, in which a federal authority participates, outside the scope of this Act, with other jurisdictions … may be taken into account in conducting an environmental assessment of a project in the region, particularly in considering any cumulative environmental effects…

Subsequently, the *Canadian Environmental Assessment Act* (2012) included provisions for the completion of “regional studies” and their consideration at various stages of subsequent project assessments, although no such studies were completed.

The current IAA contains provisions for the conduct and use of RAs, including that the federal Minister of the Environment and Climate Change (ECCC):

92 … [M]ay establish a committee — or authorize the [IA] Agency [of Canada] — to conduct a regional assessment of the effects of existing or future physical activities carried out in a region.

The remainder of the IAA’s RA-related content (Sections 92-94 and 96-103) deals with general governance and procedural matters, as well as requiring that completed RAs be considered in determining if an IA is required for a proposed project, and in conducting any such assessment (Sections 16(2) and 22(1)). A key stated objective of both RAs and SAs under the IAA is “to improve the effectiveness and efficiency of impact assessments by providing information and analysis’ (IAAC Impact Assessment Agency of Canada 2024a: 2).

**Fig. 1:**
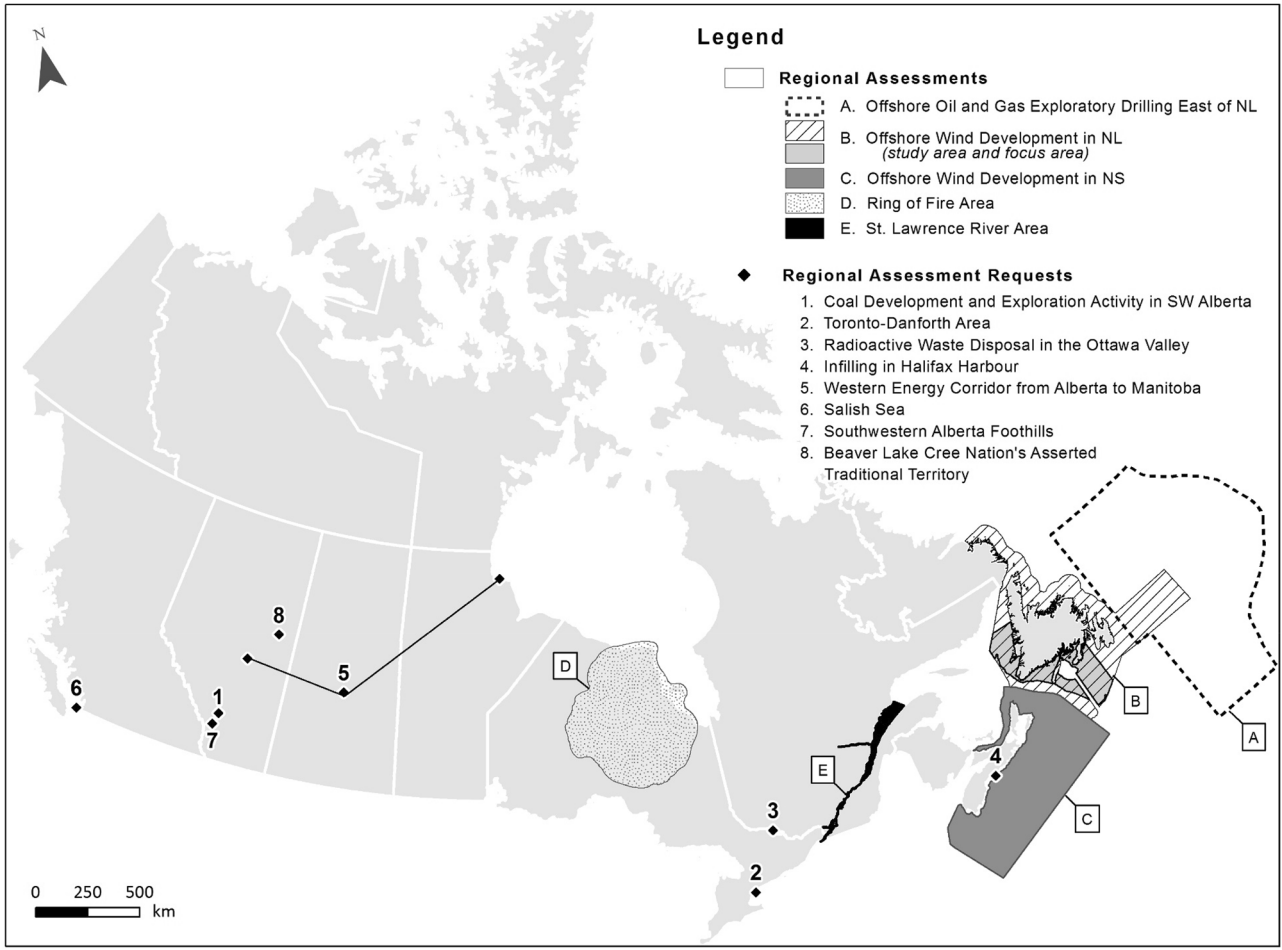
Completed, commenced and requested regional assessments under the IAA to date

As of the time of writing there have been five RAs initiated, with three completed, one in progress, and one in the planning stage (Fig. 1, Table 2)^4^. These are described briefly below, with a focus on their respective objectives and intended outcomes. The review of documentation for this analysis has focused on publicly available materials that set out the goals and planned outputs of these assessments, including government announcements of their initiation and commencement, associated agreements and terms of reference (TOR), as well as the resulting RA reports for the three completed RAs (IAAC Impact Assessment Agency of Canada 2025). All such documents are cited directly in the sections that follow, and included in the references section at the end of this paper.

**Table 2:**
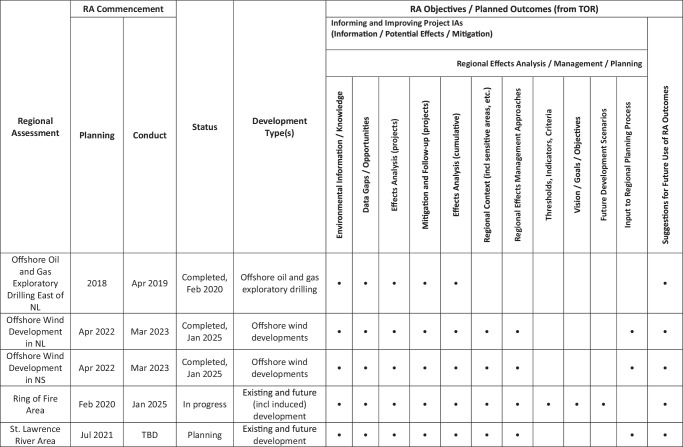
Overview of regional assessments completed or in progress under the IAA

#### Offshore Oil and Gas Exploratory Drilling East of Newfoundland and Labrador

In April 2019, the Governments of Canada and Newfoundland and Labrador announced (CA and NL Governments of Canada and Newfoundland and Labrador 2019a) an agreement and associated TOR (CA and NL Governments of Canada and Newfoundland and Labrador 2019b) to conduct a RA of offshore oil and gas exploratory drilling activities off the east coast of the Island of Newfoundland (Fig. 1). This was the first RA undertaken under Canadian federal IA legislation, and had the stated purpose of “improving the efficiency of the environmental assessment process as it applies to oil and gas exploration drilling while at the same time ensuring the highest standards of environmental protection continue to be applied and maintained” (CA and NL Governments of Canada and Newfoundland and Labrador 2019b): 2). Prior to this, individual drilling projects were subject to lengthy but extremely duplicative IAs, with the RA intended as a generic but comprehensive assessment of these activities and their effects to help define environmental requirements for future drilling in this region (Bangay et al. 2020).

The RA was completed by a five-member committee that carried out information gathering, analysis and Indigenous and public engagement over an approximately one year period, with its final report submitted in early 2020 (Bangay et al. 2020). In accordance with its TOR (CA and NL Governments of Canada and Newfoundland and Labrador 2019b), the RA provided environmental baseline information for the study area, including an associated geographic information system (GIS) application, as well as a review and analysis of the potential effects of offshore drilling and associated activities, and of mitigation and monitoring requirements for such projects. The committee made 41 recommendations to governments and other parties on a variety of topics, including: additional research to address data gaps; future updates to RA information and analysis; and mitigation and follow-up requirements for future drilling activities, including additions to those defined through previous project IAs. A considerable focus was on determining whether certain locations should be closed to drilling for environmental reasons, but in the end it was determined that there was no basis to recommend any such exclusion areas (Bangay et al. 2020).

The RA helped inform a regulation under the IAA that was brought into force in June 2020, which sets out requirements for future exploratory drilling activities in the RA study area. Under that regulation, proposed drilling projects that demonstrate conformance with these conditions are no longer subject to federal IA review requirements (IAAC Impact Assessment Agency of Canada 2020a). An RA follow-up program (CA and NL Governments of Canada and Newfoundland and Labrador 2021a) was also initiated to track implementation of the RA recommendations accepted by governments (CA and NL Governments of Canada and Newfoundland and Labrador 2020; CA and NL Governments of Canada and Newfoundland and Labrador 2021b), and to review and update RA information and analysis as needed.

#### Offshore Wind Development in Newfoundland and Labrador and Nova Scotia

In April 2022, the federal Minister of ECCC announced that a RA of offshore wind development would be carried out in two marine areas of Atlantic Canada, and directed the Impact Assessment Agency of Canada (IAAC) to engage provincial governments, Indigenous groups and others in its planning (IAAC Impact Assessment Agency of Canada 2022). This eventually resulted in federal-provincial agreements for two separate RAs covering areas offshore Newfoundland and Labrador (NL) and Nova Scotia (NS) (Fig. 1), and the appointment of two five-member committees in March 2023 to conduct these assessments under essentially identical TOR (CA and NL Governments of Canada and Newfoundland and Labrador 2023a; CA and NL Governments of Canada and Newfoundland and Labrador 2023b; CA and NS Governments of Canada and Nova Scotia 2023a; CA and NS Governments of Canada and Nova Scotia 2023b).

In parallel with RA planning and conduct, the federal and provincial governments established new joint management regimes for offshore wind development in each of these jurisdictions (NRCan Natural Resources Canada 2022a; NRCan Natural Resources Canada 2022b; NRCan Natural Resources Canada 2023). These involved amendments to the existing *Atlantic Accord Acts* that established joint federal-provincial management of the offshore oil and gas sectors in NL and NS in the mid-1980s, and an associated expansion of the regulatory mandates of the current offshore petroleum boards in each province to include marine renewables (NRCan Natural Resources Canada 2022a; NRCan Natural Resources Canada 2022b; NRCan Natural Resources Canada 2023). Regional environmental analysis and engagement conducted through the RAs were intended to help inform the selection of areas for future calls for bids and associated licencing rounds for offshore wind developments by these two offshore regulators. Given the lack of previous project development and IA experience in this sector in Atlantic Canada, the RAs were also seen as an opportunity conduct early analyses and engagement regarding these activities, their potential effects and possible mitigation (including lessons from other jurisdictions), in order to have this information available for future project assessments (CA and NL Governments of Canada and Newfoundland and Labrador 2023a; CA and NS Governments of Canada and Nova Scotia 2023a).

The conduct of both RAs commenced in March 2023 (CA and NL Governments of Canada and Newfoundland and Labrador 2023b; CA and NS Governments of Canada and Nova Scotia 2023a), with final RA reports submitted in January 2025 (Daborn et al. 2025; McDonald et al. 2025). These provide:

- generic descriptions of offshore wind developments and associated activities (construction, operations, decommissioning, potential accidental events);
- an overview of the information and analysis used to complete the assessment, and of the nature and outcomes of its associated public and Indigenous engagement activities;
- descriptions of the existing environments of their respective RA study areas (physical, biological, socioeconomic);
- an environmental constraints/mapping exercise to identify and recommend areas for future offshore wind licensing (based on existing and available information, engagement, and RA-derived geospatial criteria);
- identification of mitigation, monitoring and other measures for addressing potential adverse effects and optimizing socioeconomic benefits from future offshore wind development; and
- recommendations for further study to address information and knowledge gaps.

Both RA committees also made recommendations about the future regulation of the offshore wind industry, including that proposed developments should not be exempt from project-specific IA reviews, with many of these RA outputs intended to help inform these future assessments (Daborn et al. 2025; McDonald et al. 2025).

The RA committees also identified portions of their respective study areas that were considered suitable for future offshore wind licensing based on environmental considerations and constraints, as well as making recommendations for both project-level measures (e.g., IA baseline data and monitoring requirements; project setback distances from marine bird colonies) and sector-wide initiatives (e.g., regional environmental studies; education and training programs; technology development and innovation funding) aimed at reducing adverse effects and maximizing benefits from future development (Daborn et al. 2025; McDonald et al. 2025).

The TOR for both RAs included considerable emphasis on cumulative effects, including the identification of locations where effect accumulation was evident or likely and potential measures to manage such effects. Key to this approach was their direct link to the future offshore wind licencing systems in both jurisdictions, and their resulting ability to influence the number and distribution of future projects (CA and NL Governments of Canada and Newfoundland and Labrador 2023a; CA and NL Governments of Canada and Newfoundland and Labrador 2023b).

#### Ring of Fire Area

The Ring of Fire is located in Northern Ontario (Fig. 1), along the western edge of the James Bay Lowland which comprises a vast expanse of intact boreal forest and wetland complexes that comprises one of the largest carbon sinks on earth. It is located entirely within Treaty 9 territory, and is home to 34 First Nations and approximately 30,000 Indigenous people. The region has been subject to extensive mineral exploration over the past two decades, with numerous mining claims covering thousands of square kilometers (ROF RA WG Ring of Fire Regional Assessment Working Group 2025). Several road developments to this currently remote area have also been proposed, and Indigenous and environmental groups have highlighted the ecological and socio-cultural importance of the region and concerns about the effects of future mining and other activities (Chetkiewicz and Lintner 2014; Johnson et al. 2020).

In Fall 2019, the Minister of ECCC received three requests for a RA in this area, and in February 2020 determined that one would be undertaken (IAAC Impact Assessment Agency of Canada 2020b). RA planning has been in progress since that time, including various iterations of discussions with the Government of Ontario, Indigenous groups and others (IAAC Impact Assessment Agency of Canada 2020b). In September 2024, an RA working group comprised of IAAC representatives and participating First Nations released draft TOR for public review, which were finalized in January 2025 (ROF RA WG Ring of Fire Regional Assessment Working Group 2025). These TOR list various objectives and planned outputs for the RA, including:

- Information and knowledge on regional environmental, health, cultural, social and economic conditions, including Indigenous rights and interests (including interconnections, information gaps and opportunities).
- An understanding of potential effects (positive and negative, including cumulative effects and potential impacts on Indigenous peoples) resulting from existing and future development (scenarios).
- Innovative ways to address negative effects and enhance benefits in a way that fosters sustainability.
- Regional context for future developments and their assessments (ecological and cultural significance; key areas; regulatory and management regimes; development objectives and scenarios; frameworks, criteria and indicators for evaluating effects).
- Suggestions on how RA findings may improve future decision-making (including IAs and other processes and initiatives).

RA conduct is currently underway, with the working group scheduled to deliver its report within 30 months of RA commencement (ROF RA WG Ring of Fire Regional Assessment Working Group 2025).

#### St. Lawrence River Area

In July 2021, the Minister of ECCC announced that an RA would be conducted in the St. Lawrence River area of Quebec (Fig. 1) (IAAC Impact Assessment Agency of Canada 2021). This RA was initiated in response to a July 2020 request from the Mohawk Council of Kahnawà:ke (MCK), and subsequent IAAC analysis and Indigenous and public engagement (IAAC Impact Assessment Agency of Canada 2021). RA planning has been in progress since that time, and in February 2025 an RA working group comprised of IAAC representatives and participating First Nations released draft TOR for public review (SLRA WG Regional Assessment of the St. Lawrence River Area Working Group 2025). These draft TOR list various objectives and planned outputs for the RA, including:

- Providing regional context for the assessment area.
- Providing an understanding of current positive and adverse effects, including cumulative effects.
- Identifying information and knowledge gaps and opportunities to address them.
- Identifying and recommending mitigation measures, follow up measures and other approaches (including priority interventions and enhancement measures).
- Describing how RA findings and recommendations could inform future planning and permitting processes in a manner that fosters sustainability and restoration and enhances IA effectiveness and efficiency.
- Conducting analysis related to diverse and vulnerable groups and making associated recommendations for future IAs.

### RA Requests

The IAA and its regulations require the Minister of ECCC to respond with reasons to any public request for a RA (or SA) within 90 days of its receipt. To date, a total of 10 RAs have been requested under the IAA (Fig. 1), with two of these (Ring of Fire and St. Lawrence River) resulting in RAs being initiated, and eight leading to a decision that the requested RA would not proceed. RA requests have often focused on concerns about the effects of past or potential development activities in a region, and a perceived need for a broader and more proactive approach to addressing these beyond project-level assessments. A variety of RA objectives and outcomes have also been suggested, including: providing regional baseline information; understanding previous or foreseeable development activities and their effects; identification of mitigation for future projects; highlighting environmental sensitivities, defining thresholds and establishing a vision for future development; proactive identification of regional issues (including cumulative effects) and effect management approaches; and contributing to regional planning (IAAC Impact Assessment Agency of Canada 2025). Further details on these RA requests, including their stated rationale and purpose, and the rationales given for government’s decisions on them, are provided as supplemental information (Online Resource 1).

An IAAC operational guide (IAAC Impact Assessment Agency of Canada 2024b) outlines the RA request process and its information requirements, and various factors that are considered in evaluating these. This includes whether the requested RA could inform future federal IA decisions (namely, whether large-scale development is anticipated in the region over the next 5–10 years that will require federal IA review), indicating a key focus on RA’s potential role in informing future project-specific assessments.

## Discussion

RAs conducted under the IAA (Fig. 1, Table 2) have included single-sector assessments focused on information gathering and effects analysis, either to streamline future IA requirements (through mitigation codification and IA exemption) or to otherwise inform and improve subsequent project assessments. In some cases they have also included linkages to larger regional planning (offshore wind licencing) processes, with the intent that RA outcomes will influence the number and location of future projects. There are also on-going multi-sectoral assessments that will inform future assessment and decision-making processes, but for which the specific nature, destination and eventual use of some of their associated outputs remain under consideration.

The following sections consider the objectives and outcomes of RA practice under the IAA thus far against those set out in the previously described theoretical literature, in order to identify any key determinants of the nature and scope of RA planning and conduct to date, as well as any related considerations or challenges in achieving these in future practice.

### Informing and Improving Project IAs (Information/Potential Effects/Mitigation)

RAs carried out under the IAA have reflected a clear focus on informing and influencing subsequent project-level assessments, with a view to improving their overall effectiveness and efficiency. The first RA completed under the IAA helped inform a regulation that exempted future exploratory drilling projects from federal IA review, given RA findings and the considerable pre-RA experience in assessing and conducting these activities. In contrast, a lack of any prior development or IA experience with offshore wind projects in Atlantic Canada saw both of those RAs recommend that future development proposals continue to require project-level assessments (Daborn et al. 2025; McDonald et al. 2025). Overall, there have been calls for government to provide greater clarity on how RAs will affect future project IA requirements, processes and decisions (Blakley et al. 2020).

Beyond potential IA exemption, RAs have also been carried out as a means of compiling environmental information and identifying potential effects and required mitigation for future projects. There are enduring questions, however, about whether and how such outputs actually transfer to and have utility in subsequent IAs (Marshall and Arts 2005; Phylip-Jones, Fischer 2015; Noble and Nwanekezie 2017), and especially, have a material effect on their eventual scope (Bonnell 2020). This includes both analytical and procedural considerations, such as the typically broad-brush nature of information and analysis at the regional or strategic levels compared to that used in project assessments (Noble 2000; Slootweg and Jones 2011; Gunn and Noble 2015), and thus the degree to which RA designs and outputs are responsive to project IA requirements (Arnold et al. 2019). Concerns have also been raised around a possible lack of awareness or incentive amongst IA regulators to purposively focus and scope project IAs in this manner (Bonnell 2020). Therefore, and while the IAA includes general requirements that RAs be considered in the screening, scoping and conduct of subsequent project assessments, this has yet to be tested in practice, and the manner and degree to which project IAs will be scoped with consideration of RA outputs has yet to be confirmed. Further research is required to investigate the degree to which IA regulators, practitioners and participants view project IA efficiency as a key goal of such assessments, as well as views on which RA outputs are most likely to be useful and influential in that regard. Research should also consider how RAs can best be designed and carried out to provide information that is useful in later project IAs, as well as to identify and address any challenges in project IA processes that may impede such scoping (Bonnell 2025).

RA practice under the IAA also highlights considerations around the potential (and required) specificity of RA outputs and recommendations, which may be an important determinant of their effect on downstream IA efficiency. While, for example, the offshore exploratory drilling RA provided specific advice on mitigation requirements for future projects (leading to these being codified in regulation), both offshore wind committees addressed this aspect of their TOR by providing lists of potential measures without explicit recommendations around their future adoption and implementation. The NL RA committee stated that while it “drew upon… expertise from the offshore wind energy sector in other jurisdictions, it would be imprudent to assume these …mitigation measures can be applied…[here] without first having on-the ground studies and monitoring of local projects (McDonald et al. 2025: 10). These RA outputs are therefore most likely to promote IA efficiency by providing an information base for future proponents and regulators to draw from or refer to as opposed to directly affecting IA scope, although it may be that comparable effort is spent in determining (and possibly debating) whether certain RA-identified measures should be implemented in a particular situation.

In addition to the current emphasis on transferring information, analysis and mitigation to project IAs, there are other possible RA outputs that would likely help improve their effectiveness but which have yet to be full explored in practice. The literature often refers to RA’s role in helping define regional sustainability criteria to guide future planning and decision-making (Harriman and No**b**le 2008; Gunn and Noble 2009a, b, 2015; Atlin and Gibson 2017). While these have been seen primarily as a basis for informing regional planning, they may also have utility in individual project assessments, including where there is no such planning mechanism in place. RA-derived sustainability criteria could, for example, be used in individual assessments to help IA participants and decision-makers evaluate the acceptability (or significance) of predicted project effects, and for helping address IAA requirements to consider “the extent to which the designated project contributes to sustainability” (Section 22(1)(h)).

### Regional Effects Analysis, Management and Planning

#### Regional Effects Analysis and Context

While it has been noted that not every RA must be focused on regional cumulative effects (Arnold et al. 2022a), the well-known difficulty of addressing these at the individual project-level has certainly been an important driver for RA interest and use (Blakley and No**b**le 2021). Experience under the IAA illustrates, however, that given the variety of RA contexts, rationales and goals, it is important that such effects be given an appropriate degree of attention in any individual assessment, based on its defined scope and objectives and commensurate to their likelihood of occurrence.

The TOR for the exploratory drilling RA (CA and NL Governments of Canada and Newfoundland and Labrador 2019b) made only brief reference to cumulative effects, presumably due to its focus on addressing project-specific effects and future IA requirements. That RA did, however, include analysis of the spatial and temporal characteristics of previous drilling activity in the region as well as predictive modeling of future (2020–2028) drilling scenarios, and used its GIS application to overlay these with other projects and activities and known environmental sensitivities. While the analysis did “not suggest a high level of spatial and temporal clustering of activity and effects in the Study Area”, the RA committee was also clearly mindful that any such effects could not be effectively managed through the project-level regulatory processes that the RA was intended to inform, but rather required earlier consideration through the offshore petroleum regulator’s licencing process (Bangay et al. 2020: 150) – which that RA did not have an established connection to.

By comparison, the TORs for the offshore wind RAs included considerable focus on cumulative effects, including various associated objectives and planned outputs stemming from their linkages to the upcoming offshore wind licencing processes. Each of these RAs provided some discussion of potential cumulative effects, including a general overview of associated concepts, the need for future project IAs to consider them, applicable government programs and initiatives, and recommendations for future study, as well as noting that cumulative effects were generally considered in each committee’s recommendations around future licensing areas (Daborn et al. 2025; McDonald et al. 2025). There was also clear recognition of, and considerable emphasis on, the difficulties associated with this RA component, including that these presented “a challenge to providing specific cumulative effects information and advice at this time” (McDonald et al. 2025: 724).

Experience under the IAA to date therefore illustrates that while RA may indeed represent an earlier and broader mechanism for assessing cumulative effects compared to project IAs, as well as (in the right planning context) for their management, many of the same analytical challenges and complexities also exist at the regional level. This reinforces the importance of giving early and adequate attention to cumulative effects in RA conduct, but also highlights the need to ensure that appropriate analytical tools are identified and made available to those conducting RAs to help enhance the quality and rigor of future assessments in that regard.

In addition, while it is important that cumulative effects continue to be considered in individual project IAs and decisions (Cooper and Sheate 2004; Duinker et al. 2013) - and RA-provided “regional context” may well support this from an analytical perspective - the transfer of information from an RA to a project IA does little to address the latter’s long-recognized inability to manage cumulative effects or other regional issues that require approaches and solutions beyond project-specific designs, mitigation and decisions.

#### Going Beyond Project-Level Mitigation

RA can provide a forum for identifying larger, “beyond project-level” approaches to addressing complex and pervasive regional issues, and for making recommendations to the appropriate responsible authorities, especially in situations of inter-jurisdictional RA cooperation. These important potential RA outputs receive relatively limited attention in the literature, however, which tends to focus on project IA influences and then jump quickly to regional planning. However, and despite observations that these RA outcomes are currently based more in theory than practice (Blakely et al. 2020), it is notable that these were key aspects of the recommendations coming from both offshore wind RAs, likely in response to TOR requirements that these assessments identify “potential approaches and measures to address regional-scale and non-project specific…effects…include[ing] potential policy, plan, program, regulatory or other initiatives by governments or other relevant parties” (CA and NL Governments of Canada and Newfoundland and Labrador 2023a: 19; CA and NS Governments of Canada and Nova Scotia 2023a: 19).

#### Influencing Regional Planning and Decision-making

RA “sits delicately at the intersection of assessment, land use planning, and policy-making” (Olagunju 2016: ii), with any ability to influence the nature and pace of development (Gunn and Noble 2009a) requiring a connection between it and the planning and decision-making processes that it is intended to inform (Noble 2010; Olagunju and Gunn 2016a, 2016b; Buse et al. 2020). RA timing must also be sensitive to key planning windows and decision points (Gunn and Noble 2009a; Fidler and No**b**le 2013), with its outcomes being translated into operational terms and clear responsibilities (Gunn and Noble 2009a; Fidler and No**b**le 2013; Pope and Young 2024), and directed to bodies with the necessary authority to act on these (Noble 2008; Doelle and Critchley 2015; Atlin and Gibson 2017; Gibson et al. 2020).

This can be challenging in practice, however, as there may be no regional planning initiative or other coordinating mechanism for an RA to inform, in which case the object of assessment^5^, and the recipients and implementers of RA outputs, may be unclear (Noble 2008, 2017; Gibson et al. 2020). Also, given Canadian jurisdictional realities, RAs conducted under federal legislation may be detached from resource management and land use planning responsibilities that rest with provinces or other jurisdictions (Gibson et al. 2010; Johnston et al. 2025). The IAA allows for federal RAs to be carried out in partnership with other jurisdictions, as evidenced by the previous offshore exploratory drilling and wind development RAs, in which case RA recommendations can be directed to the relevant authorities and processes (Powell 2015; Sinclair et al. 2018; Gibson et al. 2020). In some situations and for various reasons, however, an RA may be conducted without direct and formal participation by other relevant jurisdictions, as is the case for the on-going Ring of Fire and St. Lawrence River RAs where the specific nature, and eventual destination and use, of certain RA outputs remain under consideration.

Experience under the IAA also shows that even where there are clear linkages between RAs and relevant planning processes, there may be uncertainties and challenges regarding their respective roles and the relationships between them. RA is likely to be most effective and influential when there is an overall, shared vision for development and environmental quality in the region (Gunn and Noble 2009a, 2009b) against which future development scenarios can be evaluated - but the source of that vision is not always apparent. While both offshore wind RAs had connections to planning (licencing) systems, neither of these exercises includes establishing overall goals for future wind development in these areas. The RAs therefore had no guiding principles against which to evaluate future development scenarios or to identify preferred (or undesirable) locations, which resulted in some participants expressing concerns with setting generic and somewhat arbitrary criteria through the RAs themselves (McDonald et al. 2025).

In addition, the recommendation of one offshore wind RA committee that government also “conduct a strategic assessment of offshore licencing areas (once those areas are determined) with the key objective of supporting a more thorough assessment of cumulative effects” (McDonald et al. 2025: 738) also reflects on-going questions around the roles and relationships of different assessment tools. While the concept of a multi-tiered assessment framework – where an RA helps generally define a regional PPP, which is then subject to an SA, followed by IAs for individual projects – has been referenced by some (Sinclair et al. 2017; Doelle and Sander 2020), in reality most RAs will be undertaken without SA/SEA processes in place between them and eventual project assessments. Moreover, by helping inform the initial selection and evaluation of potential licence areas for future offshore wind developments, these RAs represented the most comprehensive and proactive approach to considering and addressing cumulative effects through planning.

Finally, in addition to having well-defined and articulated goals, it is equally important that the intended destination and implementation mechanism for RA outcomes be clearly identified at the onset, and there are likely to be challenges in taking a wait-and-see approach or requiring those conducting an RA to determine how, where and by whom its outputs will eventually be used. Clarity and transparency around the use of RA outputs by decision-makers is also required. As one illustrative example, the exploratory drilling RA committee’s (quite general) recommendation that RA information be considered in future licensing decisions led to equally general responses from the offshore regulator, who committed to do so “in its land tenure decisions on a go-forward basis” (CA and NL Governments of Canada and Newfoundland and Labrador 2020: 20) and later reported that this had occurred (CA and NL Governments of Canada and Newfoundland and Labrador 2021b), without detailing whether or how the RA information was actually influential in such decisions.

## Summary and Conclusion

RA experience under the IAA to date reflects a variety of objectives and intended outcomes, as well as providing some useful lessons relevant to future RA planning and conduct.

A key focus has been on improving the effectiveness and efficiency of subsequent project-specific IAs, though the provision of RA-derived information, analysis, mitigation and regional context to these later assessments. These goals and outputs have been well reflected in the assessments undertaken thus far, as well as in RA request decisions which often hinge on whether there are foreseeable projects in the region in question that would be subject to federal IA requirements. This is perhaps understandable, given that these RAs have their most direct (and statutory) connection to federal project IAs. The degree to which RA outputs will transfer to project assessments, and materially affect their scope and conduct, remains to seen however, and experience elsewhere suggests both analytical and procedural challenges in that regard. Further research is therefore required to help future RA designs maximize the utility of their outputs at the project level, and to identify and address any challenges with doing so in, for example, the development of project IA guidelines (or TOR). There are also other potential RA outputs that may be useful in project IAs, including sustainability criteria for future development which, even in the absence of an existing or connected regional planning mechanism, can be useful in evaluating future projects and their effects.

Notwithstanding a focus on informing and influencing subsequent project IAs, it is important to reiterate that RAs and other such tools were introduced in response to the recognized limitations of project-level assessments themselves. The transfer of information from an RA to a project IA may well help improve its overall effectiveness and efficiency, but is unlikely to give it the ability to address the very issues that required a more proactive, regional approach in the first place. Indeed, RA is increasingly being viewed as an integration of assessment and planning activities (Gunn and Noble 2009a; Arnold et al. 2022a), including its potential role in informing regional planning or otherwise influencing the nature, intensity and distribution of future development. Experience suggests, however, that this can be challenging as there may be no such planning mechanism for RA to engage with, and especially, given Canadian jurisdictional realities. RAs undertaken under the IAA are therefore likely to be most successful in that regard where they are planned and conducted in cooperation with other applicable parties (Gibson et al. 2020), and have an established link to relevant planning processes. RA practice also shows, however, that even when this is the case, there may be challenges where neither exercise sets out an overall vision for future development to guide RA analysis and eventual decision-making, and due to a lack of specificity in RA outputs and the manner in which they will be or have been used in decision-making.

Joint planning and conduct of RAs amongst jurisdictions and with other partners can be a significant and often long-term undertaking, as evidenced by the Ring of Fire and St. Lawrence River RAs. Given this, and the earlier described range of potential RA goals and outputs, the planning and conduct of future RAs under the IAA could in some cases be approached in a “phased” manner. This could involve, for example, an initial RA stage focused on environmental information, analysis and engagement to identify key issues and requirements regarding future development, along with parallel discussions between governments and other parties regarding the necessity, and if so the objectives, of a subsequent RA phase which may involve additional, “higher order” RA outcomes, as applicable.

## Supplementary information

Supplemental information

## Data Availability

No datasets were generated or analysed during the current study.

## Footnotes

1 In keeping with Canadian federal legislation, this paper uses “impact assessment” to refer to the assessment process and “effect” when referring to the potential environmental, health, social and economic changes that are assessed.

2 The IAA uses the term “strategic assessment”. This terminology – as opposed to SEA – is used in this legislation in part to distinguish between these assessments and those carried out a separate 2010 Cabinet Directive that required federal departments to conduct SEAs of PPP proposals submitted for ministerial or cabinet approval.

3 Other aspects of RA design and conduct, including matters of governance, administration and Indigenous and public involvement, are typically determined on an individual RA basis based on context-specific factors and guided by overall procedures and principles set out in the IAA.

4 Supplemental information included with this paper provides additional details on RA goals and objectives (from their TOR) and on the various RA requests received to date (see Online Resource 1).

5 The term “regional SEA” (or R-SEA) has been used by some to refer to SEAs that focus on a regional PPP (CCME 2009; Gunn and Noble 2009a, 2009b; Fidler and Noble 2013).
