# Supplementary material for: Regional Assessments Under the Canadian Impact Assessment Act: Objectives, Outcomes and Lessons So Far
Source: Environ Manage. 2025 May 14;75(11):2994–3009. doi: 10.1007/s00267-025-02176-4 (PMC12546286; doi:10.1007/s00267-025-02176-4)
Supplement: Supplementary file 1 — Supplemental information [file 267_2025_2176_MOESM1_ESM.pdf]

Regional Assessments Under the Canadian *Impact Assessment Act:* Objectives, Outcomes and Lessons So Far

Supplemental Information

Table 1: Goals and Objectives of Regional Assessments (RAs) Completed or in Progress Under the *Impact Assessment Act* (IAA) (as of March 31, 2025)

<table>
<tr><th rowspan="2">Regional Assessment</th><th>Purpose / Goal</th><th>Objectives</th><th rowspan="2">Reference (See references section in main paper)</th></tr>
<tr><th colspan="2">NOTE: The text provided below is extracted and provided verbatim from the relevant RA Terms of Reference</th></tr>
<tr><td>Offshore Oil and Gas Exploratory Drilling East of NL</td><td>[I]mproving the efficiency of the environmental assessment process as it applies to oil and gas exploration drilling while at the same time ensuring the highest standards of environmental protection continue to be applied and maintained. (p. 2)</td><td>No specific “objectives” defined (and labeled as such) in the RA TOR</td><td>CA and NL (2019b)</td></tr>
<tr><td>Offshore Wind Development in NL</td><td rowspan="2">To provide information, knowledge and analysis regarding future offshore wind development activities in the Study Area and their potential effects, in order to inform and improve future planning, licencing and impact assessment processes for these activities in a way that helps protect the environment and health, social and economic conditions while also creating opportunities for sustainable economic development. (p. 4)</td><td rowspan="2">a) Providing information, knowledge and analysis related to environmental, health, social and economic conditions and the potential effects (including cumulative effects) of offshore wind development activities in the Study Area, with consideration of Indigenous knowledge, Community knowledge and scientific information throughout.
b) Providing an understanding of the regional context that can be used in considering and evaluating the potential effects (including cumulative effects) of future offshore wind development activities to inform future planning and licencing processes and impact assessments.
c) Identifying and recommending mitigation and follow-up measures and other approaches for addressing potential positive and adverse effects (both project-specific and cumulative) as part of future decision-making for offshore wind development activities.
d) Describing how the findings or recommendations of the Regional Assessment could be used to inform future planning and licencing processes for these activities in a manner that fosters sustainability and enhances the effectiveness and efficiency of their impact assessments. (p. 4)</td><td>CA and NL (2023a)</td></tr>
<tr><td>Offshore Wind Development in NS</td><td>CA and NS (2023a)</td></tr>
<tr><td>Ring of Fire Area</td><td>The purpose of the Regional Assessment is to provide information on key priorities of importance to the First Nation Partners and the Government of Canada, in the assessment area, and provide an analysis of the potential positive and negative effects, including cumulative and</td><td>The objectives of the Regional Assessment are:
a) Gathering and providing information and knowledge related to environmental, health, cultural, social and economic conditions and their interconnections, as well as Aboriginal and Treaty rights, claims and interests, at the regional scale:</td><td>ROF RA WG (2025)</td></tr>
</table>

Regional Assessment	Purpose / Goal	Objectives	Reference (*See references section in main paper*)
	NOTE: The text provided below is extracted and provided verbatim from the relevant RA Terms of Reference		
	interactive effects due to possible development activities throughout the assessment area. The Regional Assessment will also Identify and provide recommendations on how the potential cumulative and other effects may best be managed to avoid adverse consequences and enhance positive ones and how (and by what governance body or bodies) the needed follow-up steps would be determined and initiated.<br><br>The Regional Assessment is intended to inform and improve the effectiveness and efficiency of future impact assessments conducted under the Impact Assessment Act and support other decision-making processes in a way that helps to:<br>• Preserve Indigenous ways of life, traditions, laws, customs and oral history;<br>• Protect and improve the environmental, health, social, cultural and economic conditions of potentially affected communities;<br>• Protect and advance Aboriginal and Treaty rights, claims and interests within the assessment area; and<br>• Create opportunities for community and regional economic equity and sustainable development (p 21)	i. Provide an inventory of existing Indigenous and western technical and scientific data and Indigenous Knowledge where possible;<br>ii. Provide a description of existing conditions, including existing community socio-economic conditions and community-identified values, interests and priorities;<br>iii. Identify key information gaps and research needs that could be undertaken to support the Regional Assessment and future impact assessments and decision making, either concurrently or as part of follow-up and monitoring work; and<br>b) Providing an understanding of potential positive and negative effects, including cumulative effects and potential impacts on Indigenous Peoples:<br>i. Identify and describe existing and potential future development activities in the development area and associated infrastructure and induced development that may occur or that has occurred within the assessment area;<br>ii. Identify and describe components, values and concerns with respect to potential effects of development on the assessment priorities;<br>iii. Describe potential positive or negative effects, including cumulative effects, on the assessment priorities resulting from existing and potential future development activities;<br>iv. Identify potential development scenarios in the assessment area, with attention to different development intensities (e.g. status quo, low, moderate, high) and timeframes, and analyze the development scenarios to understand the potential impacts from various intensities of development on the assessment priorities; and<br>v. Identify and describe past, present and reasonably foreseeable development activities in the assessment area that could contribute to cumulative effects<br>c) Identifying innovative ways to avoid, reduce or eliminate negative effects and enhance positive effects of development in a way that fosters sustainability:	

Regional Assessment	Purpose / Goal	Objectives	Reference (*See references section in main paper*)
	NOTE: The text provided below is extracted and provided verbatim from the relevant RA Terms of Reference		
		i. Identify ways to avoid, reduce, or eliminate negative effects or maximize the positive effects of development activities, including maximizing wellbeing and economic opportunities for Indigenous Peoples and communities; and<br>ii. Identify ways to inform planning and decision-making for future development activities, in a manner that contributes positively towards sustainability and protects sensitive areas, vulnerable populations and Aboriginal and Treaty rights, claims and interests.<br>d) Providing regional context for future developments and their assessments:<br>i. Provide a description of the ecological and cultural significance of the region and its people;<br>ii. Provide a description of areas of sensitivity or value-rich geographical/ecological zones;<br>iii. Describe the existing regulatory framework and identify areas for enhanced decision making for Indigenous Peoples;<br>iv. Identify potential regional and community development objectives, outline and evaluate development scenarios and potential management regimes, including governance models that abide by community protocols and decision making, to achieve positive development outcomes; and<br>v. Develop and recommend a framework for considering and evaluating the effects of future development activities and scenarios (especially with respect to cumulative effects and ecological integrity), including criteria or indicators that may be used to evaluate the immediate and long-term effects of development.<br>e) Describing how the findings of the Regional Assessment should be considered and implemented to enhance the effectiveness and efficiency of future decision-making processes:<br>i. Describe how the findings of the Regional Assessment should be used to enhance the effectiveness and efficiency of future impact and cumulative effects assessments and	

<table>
<tr><th rowspan="2">Regional Assessment</th><th>Purpose / Goal</th><th>Objectives</th><th rowspan="2">Reference
(See references section in main paper)</th></tr>
<tr><th colspan="2">NOTE: The text provided below is extracted and
provided verbatim from the relevant RA Terms of Reference</th></tr>
<tr><td></td><td></td><td>various decision-making and monitoring or management processes; and
ii. Describe how the findings of the Regional Assessment should be used to inform regional and community initiatives, including governance and management. (p. 23 – 25)</td><td></td></tr>
<tr><td>St. Lawrence River Area</td><td>The main goal of this Regional Assessment is to gain a deeper insight into the state of the St. Lawerence River within the assessment area by assessing the relationships between targeted physical activities, complementary activities, and the ecosystems within which they operate. In this way, a set of baseline and targeted conditions, which take into account both Indigenous knowledge and modern Western science, can be established to better evaluate the effects of activities. This set of conditions will help to identify strategies for these activities and other efforts to achieve environmental and social gains that improve upon present day conditions in an efficient, collaborative manner. (p 4)</td><td>Objectives
a. Provide regional context for the assessment area.
• Describe spatial and temporal scope defined in section 4;
• Describe assessment priorities defined in section 4;
• Identify high ecological and cultural value locations currently supporting / sustaining assessment priorities.
• Describe targeted physical activities and complementary activities and their evolution over time.
• Provide an understanding of the organization of the marine transportation sector in the assessment area and give a thorough overview of the relationships between the current facilities.
• Compile, and review available data, information and knowledge to characterize and establish baseline conditions of assessment priorities in the assessment area or study zones;
• For each Indigenous community that may be affected by targeted physical activities and complementary activities, establish targeted conditions / portraits of land-marine uses and rights practiced;
    ○ To take into account their unique context, each community will be solicited to develop their respective targeted conditions.
b. Provide an understanding of current positive and adverse effects of targeted physical activities and complementary activities on environmental, health, social, cultural and economic assessment priorities.
• Compile and describe existing information (current knowledge and scientific literature) related to the effects, both positive and adverse, of targeted physical activities and complementary activities in the assessment area on the assessment priorities</td><td>SLRA WG (2025)</td></tr>
</table>

Regional Assessment	Purpose / Goal	Objectives	Reference (*See references section in main paper*)
	NOTE: The text provided below is extracted and provided verbatim from the relevant RA Terms of Reference		
		described in section 4 and on Indigenous peoples, including on the rights of Indigenous peoples recognized and affirmed by section 35 of the Constitution Act, 1982 and the United Nations Declaration on the Rights of Indigenous Peoples;<br>• Describe potential effects that may result from malfunctions and accidents related to targeted physical activities.<br>c. Provide an understanding of cumulative effects in the region.<br>• Provide an overview of foreseeable and potential future targeted physical activities, including their expected locations in the assessment area.<br>• Provide a description of areas already identified as highly affected by cumulative effects.<br>• Identify and analyze potential effects due to the targeted physical activities in combination with the effects of complementary activities (to be selected by the Regional Assessment Working Group as appropriate) that will or may affect the assessment priorities:<br>o Select the spatial and temporal boundaries of study zones appropriate for each assessment priority;<br>o Describe existing and potential cumulative effects on the assessment priorities caused by the interactions of human activities and natural processes that accumulate over time and space;<br>o Describe how climate changes can influence cumulative effects; and<br>o Based on the analysis of cumulative effects, identify any assessment priorities and locations that may be of concern / may be below targeted conditions related to these effects.<br>o Identify high ecological and cultural value locations currently supporting / sustaining assessment priorities.<br>d. Identify information and knowledge gaps and opportunities to address them.<br>• Identify and analyze information, data and knowledge gaps, focusing on assessment priorities, and any related gaps that	

Regional Assessment	Purpose / Goal	Objectives	Reference (*See references section in main paper*)
	NOTE: The text provided below is extracted and provided verbatim from the relevant RA Terms of Reference		
		may hinder the identification of baseline conditions or the conduct of impact assessments of the targeted physical activities in the assessment area.<br>• Make recommendations on the methods to fill information and knowledge gaps, to improve the effectiveness and efficiency of impact assessments in the assessment area.<br>e. Identify and recommend mitigation measures, follow up measures and other approaches including priority interventions and enhancement measures that would benefit assessment priorities<br>• Provide an overview of the different successes and lessons learned from other jurisdictions in the implementation of mitigation measures aimed at preventing or reducing effects of similar physical activities.<br>• Recommend mitigation measures or approaches that are technically and economically feasible which would address potential adverse effects of future targeted physical activities in the assessment area.<br>• Recommend other feasible measures such as:<br>o priority interventions that could have a strong, positive impact on achieving baseline conditions and/or targeted conditions;<br>o enhancement measures to create and maximize potential positive effects of targeted physical activities in the assessment area; and<br>o precautionary measures that can be applied to address concerns associated with gaps (e.g. additional studies recommended).<br>Note that even if the Regional Assessment may highlight how complementary activities contribute to cumulative effects in the assessment area, and how they may hinder the achievement of targeted conditions, it cannot make recommendations regarding complementary activities. This ensures that the Terms of Reference comply with the requirements of the Impact Assessment Act and respect federal-provincial jurisdiction.	

Regional Assessment	Purpose / Goal	Objectives	Reference (*See references section in main paper*)
	NOTE: The text provided below is extracted and provided verbatim from the relevant RA Terms of Reference		
		f. Describe how the findings and recommendations of the Regional Assessment could inform future planning and permitting processes for targeted physical activities in a manner that fosters sustainability and restoration and enhances the effectiveness and efficiency of their impact assessments.<br>• Describe how to consider, implement or otherwise address the Regional Assessment findings and recommendations to enhance the effectiveness and efficiency of future project-based impact assessments in the assessment area as well as other decision-making processes.<br>• Share knowledge and data gathered as well as findings of the regional assessment to support the collection of information for project-level assessments and inform scoping.<br>• Establish approaches for proponents when undertaking project-based impact assessments to contribute to achieving baseline and targeted conditions, with special consideration of priority interventions and enhancement measures identified through the Regional Assessment.<br>• If a follow-up program is recommended by the Regional Assessment Working Group, that program should at a minimum, consider and incorporate any new or updated information that becomes available after submission of the Report, to help ensure that the Regional Assessment remains current.<br>g. Gender-based Analysis Plus<br>• The Regional Assessment Working Group will assess gender-based analysis plus considerations (i.e., diverse and vulnerable population groups) and make recommendations on the way future impact assessment should account for and address these considerations. (p 4-7)	

Table 2: Regional Assessment (RA) Requests Under the *Impact Assessment Act* (IAA) (as of March 31, 2025)

Requested RA	Requestor and Date	Summary of RA Rationale / Proposed Objectives	Decision Date and Outcome	Decision Rationale
RA in the Ring of Fire Area	Aroland First Nation, Oct 1 2019<br><br>Wildlife Conservation Society of Canada, Nov 12 2019<br><br>Osgoode Environmental Justice and Sustainability Clinic, Dec 20 2019	• Environmental and cultural importance and sensitivity of the region.<br>• Anticipated high level of future development interest and activity once the proposed roads are built.<br>• Concern about potential effects of future mining and other human activity in the region on ecological and cultural values.<br>• Need for a proactive, comprehensive, regionally focused and participatory approach.<br>• Regional baseline data, addressing regional issues prior to project IAs, open and participative process for evaluating alternatives and developing a preferred vision for how any new development should be planned and implemented.	Feb 10 2020<br><br>RA to proceed	• Current and foreseeable proposals for roads, mining and other activities that may be subject to federal IA.<br>• Development may cause effects on areas of federal jurisdiction and impacts on Indigenous peoples (incl cumulative effects).<br>• Public interest.
RA of the St. Lawrence River Area	Mohawk Council of Kahnawà:ke, Jul 29 2020	• Concerns with increased shipping and industrialization in the river and the effects of these activities.<br>• Need for a more comprehensive regional approach to assessing the current state of the river and the cumulative effects of past, current and future activities.<br>• Impacts to the rights and interests of the Mohawks of Kahnawà:ke have already occurred.<br>• Focus on efforts to improve the current state of the environment and address past and on-going effects.	Oct 27 2020: Further analysis and engagement<br><br>Jul 15 2021: RA to proceed	• Current and foreseeable proposals for port infrastructure and other physical activities that may be subject to federal IA.<br>• Development may cause effects on areas of federal jurisdiction and impacts on Indigenous peoples (incl cumulative effects).<br>• Opportunities for collaboration.<br>• Public interest.
RA of Coal Development and Exploration Activity in Southwest Alberta	Member of Parliament through petition to Minister of ECCC, Mar 22 2021	• Concerns about the effects of proposed coal developments and exploratory activity in this region on Treaty and Aboriginal rights, water quality, species at risk and the general environment.	Jun 16 2021<br><br>RA not to proceed	• Existing regulatory frameworks and ongoing planning and policy initiatives to address effects.<br>• Various new and forthcoming policy, legislative and regulatory initiatives to reduce coal development and use.

Requested RA	Requestor and Date	Summary of RA Rationale / Proposed Objectives	Decision Date and Outcome	Decision Rationale
RA of the Toronto-Danforth Area, Ontario	Multiple individual requests, Apr-May 2021	• To assess the effects (including cumulative effects) of multiple large transit developments, climate change adaptation / infrastructure projects and other activities on the environmentally sensitive ravine system and flood zones of the Don River.	Jul 19 2021<br><br>RA not to proceed	• Existing programs and applicable regulatory requirements for proposed developments.
RA of Radioactive Waste Disposal in the Ottawa Valley, Ontario	City of Ottawa, May 3 2021	• Concerns around the potential environmental, social, economic and health effects of planned radioactive disposal projects in the region.	Jul 30 2021<br><br>RA not to proceed	• Existing legislative and regulatory instruments and policy initiatives, including project IAs, licensing, and monitoring requirements.<br>• RA results would not be available in time to inform on-going IAs.
RA of Infilling in Halifax Harbour, Nova Scotia	Various individuals and organizations, May 27 2021	• Concerns about the effects of water lot infilling in Northwest Arm on the natural environment and other users.<br>• To determine if infilling poses a cumulative threat to fish and fish habitat and other ecological values.<br>• To address the current jurisdictional and regulatory gap related to these activities.	Nov 29 2021<br><br>RA not to proceed	• RA not intended as a means of addressing jurisdictional or regulatory gaps.<br>• As infilling is not subject to federal IA, an RA is unlikely to inform future assessments.
RA of a Western Energy Corridor from Alberta to Manitoba	Western Energy Corridor Inc, Mar 23 2021 and Aug 18 2022	• RA of a potential energy corridor for future pipelines, high voltage power lines and other linear infrastructure, extending approximately 1,500 km from east central Alberta to Hudson Bay.<br>• Early analysis to determine if development within this corridor can be undertaken without causing significant effects, and to attempt to identify and resolve any issues early to help advance future projects.<br>• Provide information to subsequent project specific IAs.	Jun 21 2021 and Nov 15 2022<br><br>RA not to proceed	• Lack of reasonably foreseeable future developments that would require federal IA.<br>• Existing regulatory frameworks and initiatives.
RA in the Salish Sea, British Columbia	Salish Sea Indigenous Guardians Association, Jun 21 2022	• Request for a phased RA approach, focused first on data collection and trend analysis, defining baselines / context, targets and thresholds and mitigation approaches, and eventually, creation of a sustainable regional development plan.	Sept 20 2022<br><br>RA not to proceed	• Numerous existing and on-going programs and initiatives.<br>• Government's on-going review of RA recommendations from Review Panel for the Roberts Bank Terminal 2 Project.

Requested RA	Requestor and Date	Summary of RA Rationale / Proposed Objectives	Decision Date and Outcome	Decision Rationale
RA in the Southwestern Alberta Foothills	Submitted by Canada Energy Regulator on behalf of various Indigenous groups, Nov 29 2022	• RA interest and suggestions stemming from recent regulatory review of the NOVA Gas Transmission Ltd. West Path Delivery 2023 Project.<br>• Potential cumulative effects on Aboriginal / Treaty rights in the region, and need for a robust baseline to inform effects management.	Feb 27 2023<br><br>RA not to proceed	• Lack of reasonably foreseeable future developments that would require federal IA.<br>• Existing regulatory frameworks and ongoing planning and policy initiatives.
RA of Beaver Lake Cree Nation's Asserted Traditional Territory, Northeastern Alberta	Beaver Lake Cree Nation, Feb 8 2024	• Environmental and cultural importance of area.<br>• Effects of past, ongoing and anticipated future development on the natural environment and exercise of traditional activities / rights.<br>• Need enhanced understanding of future development and past and potential effects, and baseline analysis of environmentally sensitive areas for future IAs.	Sept 16 2024<br><br>RA not to proceed	• Lack of reasonably foreseeable future developments that would require federal IA.<br>• RA results would not be available in time to inform on-going IAs.<br>• Cumulative effects must be considered in project IAs.<br>• Presence of existing regulatory and policy frameworks and land use planning processes.
Source: Information summarized from RA request documents available at:<br>Canadian Impact Assessment Registry. https://iaac-aeic.gc.ca/050/evaluations/index?culture=en-CA<br>(Accessed March 31 2025)				
